# Automated facial expression analysis of participants self-criticising via the two-chair technique: exploring facial behavioral markers of self-criticism

**DOI:** 10.3389/fpsyg.2023.1138916

**Published:** 2023-04-25

**Authors:** Júlia Halamová, Martin Kanovský, Guilherme Brockington, Bronislava Strnádelová

**Affiliations:** ^1^Faculty of Social and Economic Sciences, Institute of Applied Psychology, Comenius University Bratislava, Bratislava, Slovakia; ^2^Faculty of Social and Economic Sciences, Institute of Social Anthropology, Comenius University Bratislava, Bratislava, Slovakia; ^3^Center for Natural and Human Sciences, Federal University of ABC, São Paulo, Brazil

**Keywords:** facial expression emotions, facial action coding system, self-criticism, two chair technique, iMotions software

## Abstract

**Introduction:**

As self-rating scales are prone to many measurement distortions, there is a growing call for more objective measures based on physiological or behavioural indicators. Self-criticism is one of the major transdiagnostic factor of all mental disorders therefore it is important to be able to distinguish what are the characteristic facial features of self-criticizing. To the best of our knowledge, there has been no automated facial emotion expression analysis of participants self-criticising via the two-chair technique. The aim of this study was to detect which action units of facial expressions were significantly more often present in participants performing self-criticism using the two-chair technique. The broader goal was to contribute to the scientific knowledge on objective behavioural descriptions of self-criticism and to provide an additional diagnostic means to the existing self-rating scales by exploring facial behavioral markers of self-criticism.

**Methods:**

The non-clinical sample consisted of 80 participants (20 men and 60 women) aged 19 years to 57 years (*M* = 23.86; SD = 5.98). In the analysis we used iMotions’s Affectiva AFFDEX module (Version 8.1) to classify the participants’ actions units from the self-criticising videos. For the statistical analysis we used a multilevel model to account for the repeated-measures design.

**Results:**

Based on the significant results the self-critical facial expression may therefore comprise the following action units: Dimpler, Lip Press, Eye Closure, Jaw Drop, and Outer Brow Raise, which are related to contempt, fear, and embarrassment or shame; and Eye Closure and Eye Widen (in rapid sequence Blink), which are a sign that highly negative stimuli are being emotionally processed.

**Discussion:**

The research study need to be further analysed using clinical samples to compare the results.

## Introduction

### Self-criticism and self-reassurance

According to Kannan and Levitt (2013, p. 166), self-criticism is ‘conscious evaluation of oneself that can be healthy and reflexive behaviour, but also can have harmful effects and consequences for an individual’. Furthermore, self-criticism is one factor in all sorts of psychiatric diagnoses (Longe et al., 2010; Kelly and Carter, 2012; Kannan and Levitt, 2013; Castilho et al., 2015), and greatly impacts on an individual’s emotions (Kramer and Pascual-Leone, 2016) and responses to all kinds of treatment (Shahar et al., 2012). Often, it is also related to perfectionism (Cheli et al., 2020). Due to the harmful effects of self-criticism and as it correlates with a wide range of psychopathology, we consider it important to look for ways to identify and than measure this construct among individuals. Afterwards, we can possibly intervene to reduce the destructive form of self-criticism as a way of dealing with self.

Research by Blatt et al. (1976) showed that self-criticism can take different forms whilst simultaneously having different functions. Along similar lines, Gilbert et al. (2004) have demonstrated the evolution and motivational function of self-criticism. As a result, scholars distinguish between different forms of self-criticism. Some people criticise themselves in the belief it will motivate them to achieve something that will make them more competent and perfect. Others are uninterested in improving parts of themselves they feel are unacceptable, and simply wish to get rid of them. The perceived function of self-criticism affects how people feel, behave, and think in relation to themselves. Based on this evolutionary model, Gilbert et al. (2004) proposed two negative forms: Inadequate Self and Hated Self. Inadequate Self contains feelings of personal inadequacy embodying experiences of failure, inadequacy, and a tendency to be critical. In hated self, the self-criticism is associated with a destructive attitude toward oneself, characterised by a desire to harm, hate, or act aggressively.

Watson et al. (1998) stated that the principal antecedent of depressive helplessness is the harsh negative affect accompanying self-criticism. Contempt for the self is viewed as a negative affect producing shame and helplessness (Greenberg and Paivio, 1997). What is also alarming is that self-critical tendencies are reflexive psychological behaviours that most people engage in Whelton and Greenberg (2005) and subsequently suffer from.

### Related work

Although emotions evidently play a role in the self-critical inner voice, there is little research analysing the different emotions and experiences that emerge during self-criticism. Whelton and Greenberg (2005) observed the self-criticising process and its immediate effect on individuals who had performed self-criticism. In that study students did an imagination exercise and then researchers videotaped the students criticising themselves and their responses to their own criticism. To measure self-criticism respondents were administered DEQ questionnaires (Depressive Experiences Questionnaire, DEQ; Blatt et al., 1976), and then SPAFF (Specific Affect Coding System, Gottman et al., 1996) was used to observe their emotions. SPAFF is used to code the behaviour of sixteen discrete emotions. The SPAFF Observational Coding of emotions showed that self-critics exhibited more contempt and disgust when self-criticising than the control group did. The emotions coding also revealed that self-critics were less self-resilient to criticism than the control group: they were more submissive, sadder, and more ashamed than the control group.

Kramer and Pascual-Leone (2016) examined the role of emotion in self-criticism focusing on respondents with anger problems. They compared anger-prone undergraduate students with a control group on the process indices of contempt, fear, shame, anger, and global distress, as well as on access to underlying need. Participants worked through personalized self-critical content using a single-session enactment from emotion-focused therapy, augmented with a standardized procedure for priming participants to focus on their unmet needs. The findings indicate that both groups reported reduced distress, fear, and shame, and increased assertive anger. In addition, anger-prone individuals generally expressed more self-contempt and had more difficulty accessing their underlying needs.

When studying descriptions of a person’s self-critical experience and differences between high and low self-critical participants in their imageries, Halamová et al. (2019) used compassionate imagery to evoke the inner critical, protective, and compassionate voice. The results showed differences in the imageries in relation to level of self-criticism. Both high and low self-critics displayed difficulties in overcoming their self-criticism. Unlike the high self-critics, the low self-critics had more constructive and positive strategies for dealing with their self-criticism. Gilbert (2010) pointed out that high-self critics found it easy to imagine the self-critical part of the self, but had difficulty showing their self-compassionate part. By contrast, low self-critics had problems recalling their self-critical part, but could easily express self-compassionate images. To sum up, it seems that when recalling their self-critical part, high-self-critics may experience a more intensive hated self-critical manifestation or description of the self-critical part and emotions such as (self)hate, shame, contempt, fear, incompetence, helplessness, and worthlessness.

However, it is not evident, which action unutis as facial markers people display when engaging in self-criticism. This is important to understand as well as the complex the self-criticising process because the way in which people speak with themselves has an effect on their mental and physical health (Zessin et al., 2015). Furthermore, the relationship between self-criticism and mental health does not seem to be a dichotomous characteristic which point out to the presence of the self-criticism or no criticism. It may depend on how much the person able to manage, control and dialogue with the critical part of the self, even transform this internal monologue (Volpato et al., 2022).

### Two-chair technique for studying emotion in self-criticism

A promising way of eliciting and enacting self-criticism is offered by the experiential two-chair technique often used in Gestalt therapy (Perls et al., 1951) and Emotion-focused therapy (Elliott et al., 2004). The two-chair technique helps the person to separate out the two opposing parts of the self: the self-critic and the experiencing self (Greenberg, 2002). The two-chair technique as one of experiential techniques like guided imagery or role-play have the power to trigger more emotions than just recalling an episode and intensely activate self-criticism related schemas (Centonze et al., 2021a,b).

The two-chair technique may increase self-compassion and self-protection, and reduce the intensity of the self-criticism (Shahar et al., 2012). Other studies have shown it can help in the treatment of attachment issues manifested in unresolved anger (Narkiss-Guez et al., 2015) or in touching in with some basic human needs like safety, love, competence, affiliation, nurturance, or identity (Pascual-Leone and Greenberg, 2007). Based on these findings, we think the two-chair technique is a stimulating way of eliciting and exploring emotions in individuals criticising themselves. To the best of our knowledge, there has been no automated facial emotion expression analysis of participants self-criticising via the two-chair technique. Similar studies have employed an observer-based rating system for various affective states (Whelton and Greenberg, 2005; Kramer and Pascual-Leone, 2016). In another study emotion measures were based on a coding scheme measuring contemptuousness of self-criticism developed for that study (Kramer and Pascual-Leone, 2016). Therefore, we think it is important to be able to detect facial emotions in self-criticising respondents using automated facial analysis and not only observer-based rating as it was done in studies of Kramer and Pascual-Leone (2016) and Whelton and Greenberg (2005).

### Aim of the study

The aim of this study was to detect the action units of the facial expressions that occur significantly more frequently in individuals self-criticising via the two-chair technique. These findings can contribute to the scientific knowledge on objective behavioural descriptions of self-criticism such as facial markers. In order to assess self-criticism in real time we have to elicit it to let the behavioral markers appear. Two-chair technique has the power to trigger emotions and activate self-criticism related schemas. This offers an option to provide an additional diagnostic means to the existing self-rating scales by exploring behavioral – facial markers of self-criticism.

As there is no previous research of automated facial analysis of action units during self-criticizing, we formulated the following research question instead of hypotheses: What are action units of the facial expressions that occur significantly more frequently during self-criticising via the two-chair technique?

## Methods

### Research sample

Our available sample, acquired through social networks as facebook, Instagram, and various public social for a such as related to health care or different hobbies groups, consisted of 80 participants, of whom 20 were men and 60 were women. The age of the participants ranged from 19 years to 57 years (*M* = 23.86; SD = 5.98). The data were collected in accordance with the ethical standards of the related institutional research committee and the 1964 Helsinki declaration and its later amendments or comparable ethical standards. The study was approved by an Ethical Committee of a related University under No. 4/2020. Written informed consent was obtained from all participants.

### Research procedure

In the research study, we created a research script to standardise the data collection. The script was inspired by the research of Whelton and Greenberg (2005) and Kramer and Pascual-Leone (2015). Like the participants in their research, our participants conducted a short self-critical dialogue with themselves using the two-chair technique while being recorded on a video camera. Upon arrival, the participants were seated at a laptop and asked to consent to the research. They were then requested to sit in one of two chairs placed 0.6 meters apart and facing each other. Two camcorders on tripods were positioned 1.5 m away from each chair. The cameras were located so participants were in shot from the shoulders upwards. The research assistant read the following instructions: *‘Make sure you are sitting comfortably. If you want, you can close your eyes to help you concentrate. Now try to remember a specific situation in your life when you did something wrong, when you failed at something, when you failed, when something failed, or even when you were dissatisfied with yourself. Remember where this situation took place … who was present … what was going on at the time … what exactly happened … what you wanted … what you felt … … what you were thinking … what you experienced … how you reacted to it. You will now have a few minutes of silence in which to remember as vividly as you can all the details of your failure. I will notify you when the time has elapsed and then when you are ready please open your eyes.’*

A 2.5 min silence followed in which the participants performed their imagination and then the instructions continued: *‘Everyone has a part of themselves that watches them, monitors them, and evaluates what they do. What we criticise ourselves for varies from person to person, but we all have our own version of this critical inner voice. Now I would like to ask you to be this critical voice of yours. Imagine you are sitting in the chair opposite you (the researcher points to the opposite chair) and say aloud to yourself what your inner self-critical voice usually says to you in a situation where you have failed. Be your critical inner voice now and talk to yourself, saying whatever, to criticise yourself. Speak to yourself in the 2nd person singular. Speak in this voice for 5 min. I’ll tell you when the time is up.’*

After reading the instructions, the researchers turned on the video camera and the participant delivered a 5-min self-critical monologue. If any of the participants was unable to sustain this process for 5 min, the research assistant provided help and encouragement by asking helpful questions such as: *‘What else do you usually say to yourself when something goes wrong? What words do are you use to criticise yourself? How do you swear at yourself? What are you blaming yourself for? What else? Anything more?’*

### Research instruments

In the analysis we used iMotions’s Affectiva AFFDEX module (Version 8.1) to classify the participants’ actions units from the self-criticising videos (iMotions, 2016). The Affectiva AFFDEX classifier from iMotions software (iMotions, 2016) provides probability-like values for 34 of the 98 actions units originally described in the Facial Action Coding System (FACS; Ekman et al., 1978/2002). The main actions units relevant to this study are: Brow Furrow, Outer Brow Raise, Cheek Raise, Chin Raise, Dimpler, Eye Closure, Eye Widen, Inner Brow Raise, Jaw Drop, Lip Corner Depressor, Lip Corner Puller, Lip Press, Lip Pucker, Lip Stretch, Lip Suck, Lid Tighten, Mouth Open, Nose Wrinkle, and Upper Lip Raise. iMotions Affectiva AFFDEX also works with Smile, defined as Lip Corner Pulling Outwards and Smirk, defined as an asymmetric lip corner pull (either on the right or left side of the face but not both).

### Data analysis

Statistical program R version 3.6.1 (R Core Team, 2019), package “lme4” (Bates et al., 2015), was used for the statistical analysis. In order to account for the repeated-measures design (observations were not independent, but grouped within individuals and action units), a multilevel model was fitted (80 respondents, 20 action units for each respondent). The multilevel model had two parameters: ID (accounting for the variability among respondents) and AU (accounting for the variability among action units). Both were treated as random effects. The response is binomial (presence/absence of the identification of an action unit, 0/1) so a logistic multilevel regression model was used (binomial family with logit link). We used absolute thresholds of 50 in line with iMotions (2016), which means that all values up to 50 were set to 1 and all values over 50 were set to 0 to create binary data. The variance of random effects (ID and AU) is reported, together with the overall effect size (conditional R^2^ measure) based on the theoretical (latent scale) value (Nakagawa and Schielzeth, 2013; Nakagawa et al., 2017).

## Results

**Figure 1:**
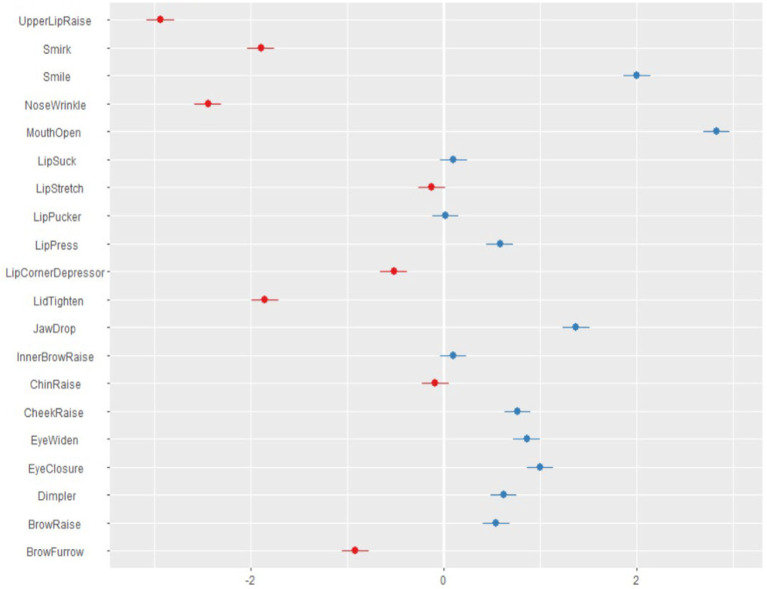
Action units of facial expressions during self-criticising.

The ID variance of the multilevel model was 0.41, and the AU variance was 2.01: therefore, variance of action units (AU) is far large than variance of respondents (ID), which means that individual differences among respondents are not as large as the differences among the action units. R^2^ is 0.42. The following action units were significantly more frequent during self-criticising using the two-chair technique: Mouth Open, Smile, Jaw Drop, Eye Closure, Eye Widen, Cheek Raise, Dimpler, Lip Press, and Outer Brow Raise. See Figure 1.

## Discussion

In this study we detected that action units of facial expressions were present significantly more often during the self-criticising part of the two-chair technique. The following action units appeared significantly more frequently: Mouth Open, Smile, Jaw Drop, Eye Closure, Eye Widen, Cheek Raise, Dimpler, Lip Press, and Outer Brow Raise.

We assume that human coders would code Eye Closure and Eye Widen as the Blink action unit as they would be unable to code them individually in real-time as the Affectiva AFFDEX classifier from the iMotions software (iMotions, 2016) can. The software creates 30 frames per second, which is impossible for humans to do. Therefore, we consider Eye Closure and Eye Widen to be the result of enhanced emotional processing elicited primarily by stimuli of negative valence (Vanman et al., 1998). Likewise, Dichter et al. (2002) found that participants blinked most when viewing negative stimuli. In the two-chair technique, participants had to talk self-critically to their imagined self in the other chair, and they clearly suffered when attacked by their own self-critic. It is unsurprising therefore that the participants significantly exhibited both more Eye Closure and more Eye Widen action units.

We found that throughout the process of self-criticising using the two-chair technique, participants displayed significant action units, and so we can guess which complex facial expressions were simultaneously present at a particular moment, and therefore which action units were grouped together The significantly more frequent action units included Dimpler, which is considered to be a sign of self-contempt (Keltner, 1995; Elfenbein et al., 2007). For some people contempt may also be linked to Outer Brow Raise or Eye Closure (Keltner, 1995). By contrast, Brow Raise, Eye Widen, and Jaw Drop are associated with fear (e.g., iMotions, 2016; Hyniewska et al., 2019), while Lip Press, Smile, and Eye closure could be related to embarrassment (Keltner, 1995; Elfenbein et al., 2007), or even shame (Izard, 1977; Elfenbein et al., 2007). All of these were significant. This is in line with previous research that found that self-critical people displayed more contempt, fear, and shame when self-criticising (Whelton and Greenberg, 2005; Gilbert (2010)). Our research seems to show that people might be generally more contemptuous, fearful, and embarrassed or shameful when being self-critical towards themselves no matter how they score on the scale of self-criticism.

The Mouth Open and Jaw Drop action units are probably significant because the video recording shows the participants’ speaking to themselves self critically which required them to open their mouths and speak. Presumably, then, the connection with Mouth Open and Jaw Drop is with the act of speaking rather than self-criticism.

We think that Smile and Cheek Raise could be attributed to the presence of the two research assistants in the room while the participant were criticising themselves. Criticising oneself is an intimate activity and the research assistants’ presence may have induced feelings of embarrassment in the participants. The smile, significant in the research study, seems to be sincere, so it may be that participants were using it to mitigate the impact of their words on themselves by smiling. This kind of smile is sometimes used to reduce physical arousal and or when restraining from emotional flooding (Gottman and Silver, 1999).

The main limitation of our work is the much larger proportion of women in the available sample, which limits the extent to which generalizations can be made.

Furthermore, a major limit of the present study is also the lack of state-emotions assessment. Such scales could have been administered after the two-chair technique to evaluate the intensity of the supposed emotions as the contempt, embarrassment or shame. In line with this, there are some versions of self-rating scale of current (state) feelings, e.g., Shame and Guilt State Scale (SGSS-8) (Cavalera et al., 2017). They are able to assess the current levels of emotions (Cavalera et al., 2018) which might be elicited by the different tasks, even after the two-chair dialogue.

Thus, future studies may address this point of incorporating the self-rating scales combining action’s unit analysis that may ensure the effective presence of the supposed state emotions. Then, trait emotions may also be significant moderator of the process (see, e.g., Harder and Greenwald, 1999). The research study also needs to address and be further analysed using clinical samples to compare the results with the non-clinical samples. In future research, we also suggest a time series of action units should be investigated to find out which action units are grouped together at one moment so we can better describe the unique facial expression of self-criticism or its potential variations.

As mentioned above, detecting facial emotions generally during a self-criticising task provides opportunities for further comparisons of groups of respondents with various levels of self-criticism. Hopefully, in the future, this will expand the scientific knowledge by providing more objective behavioural descriptions of self-criticism, thereby enabling better diagnosis and greater knowledge of the distinct facial expressions of individuals as the current use of self-rating scales is prone to all sorts of biases.

## Conclusion

This study found that the self-critical facial expression may consist of the Dimpler, Lip Press, Eye Closure, Jaw Drop, and Outer Brow Raise action units, which are related to contempt, fear, and embarrassment or shame, and that Eye Closure and Eye Widen (in rapid sequence Blink) may be a sign that highly negative stimuli are being emotionally processed.

## Data availability statement

The raw data supporting the conclusions of this article will be made available by the authors, without undue reservation.

## Ethics statement

The studies involving human participants were reviewed and approved by Ethical Committee of Faculty of Social and Economic Sciences at Comenius University in Bratislava. The patients/participants provided their written informed consent to participate in this study. Written informed consent was obtained from the individual(s) for the publication of any potentially identifiable images or data included in this article.

## Author contributions

JH designed research project. GB processed data. MK performed the statistical analysis. All authors wrote the first draft of the article, interpreted the results, revised the manuscript, and read and approved the final manuscript.

## Funding

Writing this work was supported by the Vedecká grantová agentúra VEGA under Grant 1/0075/19. This work was supported by the Slovak Research and Development Agency under the Contract no. PP-COVID-20-0074.

## Conflict of interest

The authors declare that the research was conducted in the absence of any commercial or financial relationships that could be construed as a potential conflict of interest.

## Publisher’s note

All claims expressed in this article are solely those of the authors and do not necessarily represent those of their affiliated organizations, or those of the publisher, the editors and the reviewers. Any product that may be evaluated in this article, or claim that may be made by its manufacturer, is not guaranteed or endorsed by the publisher.
